# Yogurt Enriched with Inulin Ameliorated Reproductive Functions and Regulated Gut Microbiota in Dehydroepiandrosterone-Induced Polycystic Ovary Syndrome Mice

**DOI:** 10.3390/nu14020279

**Published:** 2022-01-10

**Authors:** Tiange Li, Yue Zhang, Jiajia Song, Lijun Chen, Min Du, Xueying Mao

**Affiliations:** 1 Key Laboratory of Functional Dairy, Ministry of Education, College of Food Science and Nutritional Engineering, China Agricultural University, Beijing 100083, China; 13126750913@163.com (T.L.); zhangyue@163.com (Y.Z.); 2 College of Food Science, Southwest University, Chongqing 400715, China; jiajias@swu.edu.cn; 3 Beijing Sanyuan Foods Co., Ltd., Beijing 100009, China; chenlijun@263.net; 4 Department of Animal Sciences, Washington State University, Pullman, WA 99164, USA; min.du@wsu.edu

**Keywords:** polycystic ovary syndrome, synbiotic yogurt, gut microbiota, bile acid, inulin

## Abstract

The effects of synbiotic yogurt supplemented with inulin on the pathological manifestations and gut microbiota–bile acid axis were investigated using a dehydroepiandrosterone (DHEA)-induced polycystic ovary syndrome (PCOS) mice model. Female C57BL/6J mice were injected subcutaneously with DHEA at a dose of 6 mg/100 g BW for 20 days to establish a PCOS mouse model. Then, the PCOS mice were treated with yogurt containing inulin (6% *w*/*w*) at 15 mL/kg BW for 24 days. Results showed that supplementation of synbiotic yogurt enriched with inulin to PCOS mice decreased the body weight gain, improved estrus cycles and ovary morphology, and reduced the levels of luteinizing hormone while increasing the levels of follicle-stimulating hormone and interleukin-22 in serum. At the genus level, synbiotic yogurt increased the relative abundance of *Lactobacillus*, *Bifidobacterium,* and *Akkermansia*. PICRUSt analysis indicated that KEGG pathways including bile acid biosynthesis were changed after inulin-enriched synbiotic yogurt supplementation. Synbiotic yogurt enriched with inulin also modulated the bile acid profiles. In conclusion, inulin-enriched synbiotic yogurt alleviated reproductive dysfunction and modulated gut microbiota and bile acid profiles in PCOS mice.

## 1. Introduction

Polycystic ovary syndrome (PCOS) is a common heterogeneous endocrine disease with a high prevalence in reproductive-aged women worldwide [1]. This disorder is the primary cause of female infertility defined by a combination of ovarian dysfunction, dysfunctional follicular maturation, and ovarian hormone dysregulation, manifesting as hyperandrogenism and the hypersecretion of luteinizing hormone (LH) [2]. PCOS patients showed an accumulation of cystic follicles, an increase in ovarian stromal thickness, and a reduction in corpus luteum, accompanied by the loose arrangement of granulosa cells in the ovary, which has been replicated in PCOS mice [2,3]. Women suffering from PCOS have an elevated risk of obesity, insulin resistance, hypertension, cardiovascular disease, and other types of metabolic dysfunction [4]. Due to the high prevalence and association with multiple metabolic diseases, controlling the development of PCOS is of great importance for the reproductive health of women.

Gut microbiota has been proven to be key requirements in maintaining host health including metabolic homeostasis, immunity, and gut barrier function [5]. Recent studies showed that the dysbiosis of gut microbiota is linked to the progression of PCOS [6]. Compared with healthy women, the gut microbial diversity was reduced in PCOS patients with the change of relative abundance of specific *Bacteroidetes* and *Firmicutes* [7,8]. Gut microbiome alteration was in relation to hyperandrogenism in women with PCOS, which implies the potential role of testosterone in the structure of gut microbiota [8]. Bile acids are cholesterol-derived endogenous metabolites produced in the liver [9]. Primary bile acids are the immediate products of cholesterol catabolism and serve as substrates for the enzymes derived from intestinal flora and then converted into secondary bile acids, which are returned to the liver by the enterohepatic cycle [10]. The connection between bile acids, gut microbiota, and metabolic disorders implies that bile acids can participate in the regulation of metabolic dysfunction associated with PCOS [11]. KEGG analysis showed that the pathways for bile acid metabolism were altered in women with PCOS [12]. The level of circulating conjugated primary bile acids was increased in PCOS individuals, which was positively correlated with hyperandrogenism [13]. These data suggest that the improvement of gut microbiota and bile acid metabolism may be a promising approach to treat PCOS.

Nutritional interventions of probiotics, prebiotics, or synbiotics are effective ways to improve the gut microbiota and metabolic diseases [14,15]. Consistently, synbiotic supplementation had favorable effects on the insulin concentrations and the level of lipid profile markers in serum of PCOS patients [16]. Probiotic supplementation modulated the inflammatory biomarkers and improved clinical and laboratory features in PCOS women [17]. As a prebiotic, inulin is a fructan-type oligosaccharide that can be fermented by intestinal bacteria, including *Bifidobacterium* and *Lactobacillus,* and stimulate the growth of probiotics [18]. Inulin can regulate the gut microbiota with the increase in *Bifidobacteria* and *Akkermansia muciniphila* abundance, as well as improving metabolic disturbance in obese or type 2 diabetic individuals [19,20]. Inulin treatment also alleviated PCOS via the inhibition of inflammation and the modulation of gut microbiota in mice [21]. Yogurts and other dairy products are rich in probiotic cultures in the diet. However, the effects of yogurts supplemented with inulin on PCOS have not been examined, and the relationship among synbiotics, gut microbiota–bile acid axis, and PCOS remain poorly understood. Therefore, the effects of yogurt containing inulin on the pathological conditions, gut microbiota, and bile acid profiles in dehydroepiandrosterone (DHEA)-induced PCOS mice were investigated in this study.

## 2. Materials and Methods

### 2.1. Animal Experiments

The animal study protocol was approved by the China Agricultural University Animal Ethics Committee (Serial Number: AW12099102). Four-week-old female C57BL/6J mice were purchased from Beijing Vital River Laboratory Animal Technology Co., Ltd. (Beijing, China). Mice had free access to food and water and were housed in a temperature-controlled room (22 ± 2 °C) under a 12:12 light–dark cycle.

After one-week acclimation, mice were randomly assigned to two groups. One group was injected subcutaneously with DHEA (6 mg/100 g BW) dissolved in soybean oil for continuous 20 days to establish an animal model of PCOS. Another group (control group, *n* = 10) was injected daily with a soybean oil vehicle. Mice in the PCOS group exhibited acyclic/irregular ovarian cyclicity. Then, the PCOS mice were assigned to three groups: model group (*n* = 10), yogurt group (*n* = 10), and synbiotic yogurt group (*n* = 10).

The yogurt group was administered with fermented yogurt (15 mL/kg BW), while the synbiotic yogurt group was administered with fermented yogurt containing inulin (6% *w*/*w*) at 15 mL/kg BW daily by gavage for consecutive 24 days. At the end of the study, mice were sacrificed. The livers and ovaries were excised for further analysis.

### 2.2. Assessment of Estrous Cycle

There are four stages of an estrous cycle—proestrus, estrous, metestrus, or diestrus. Morphometric analysis of vaginal epithelial cells was conducted to determine the estrous cycle. The stage of the estrous cycle was assessed on account of the relative ratio of cornified epithelial cells, leukocytes, and nucleated epithelial cells stained in the vaginal smear. The ddH2O was pipetted gently over the vaginal opening and flushed several times to collect vaginal cells. The final flush was moved to a glass slide, allowed to dry, stained by Wright–Giemsa stain (Beyotime, China) for 5 min, and then visualized using light microscopy.

### 2.3. Measurement of Hormones

The blood samples were centrifuged at 1000× *g* for 15 min at 4 °C to collect serum and then kept at −20 °C until analysis. Progesterone (PROG), estradiol (E2), total testosterone (T), follicle-stimulating hormone (FSH), luteinizing hormone (LH), and prolactin (PRO) were measured using corresponding ELISA kits (Cloud-Clone Corp, Houston, TX, USA).

### 2.4. Hematoxylin–Eosin (H&E) Staining

Ovarian specimens were separated and then fixed with 4% paraformaldehyde solution. The samples were then dehydrated, embedded in paraffin, which was stained with H&E staining, and mounted on a glass slide. The amounts of cystic follicle and corpora lutea were morphologically examined using the Olympus light microscope to inspect the changes in the ovary. Cystic follicles had a thin layer of theca cells and granulosa cells arranged closely [21].

### 2.5. Sequencing and Analysis of Gut Microbiota

At the end of the protocol, fresh fecal samples were collected separately and stored at −80 °C. Bacterial DNA was extracted using TruSeqTM DNA Sample Prep Kit. The DNA purity and concentration were measured by NanoDrop2000 Spectrophotometer, and the agarose gel electrophoresis was used to check the integrity of DNA. The variable region V3–V4 of the 16S rRNA gene was amplified with primers 338F (5′-ACTCCTACGGGAGGCAGCAG-3′) and 806R (5′-GGACTACHVGGGTWTCTAAT-3′). The PCR reactions were conducted as 20 mL mixtures containing 4 mL 5 FastPfu Buffer, 2 mL 2.5 mM dNTPs, 0.8 mL each primer (5 mM), 0.4 mL of FastPfu Polymerase, 0.2 mL BSA, and 10 ng of template DNA. The PCR products were separated on a 2% agarose gel and purified by the AxyPrep DNA Gel Extraction Kit. The TruSeqTM DNA Sample Prep Kit was used to construct the Miseq library, and sample libraries were pooled and sequenced on an Illumina MiSeq platform according to the established protocols of Majorbio Bio-Pharm Technology Co., Ltd. (Shanghai, China).

The processing and bioinformatics analyses of the raw data were performed as previously described [22]. Raw fastq files were analyzed by using QIIME (version 1.9.1) software, and the reads that could not be assembled were deleted [23]. Sequencing analyses were performed by Uparse software (version 7.1, http://drive5.com/uparse/, accessed on 1 December 2021), and operational taxonomic units (OTUs) were clustered at a 97% similarity level. Chimeric sequences were identified and removed with UCHIME (http://www.drive5.com/uchime/uchime_download.html, accessed on 1 December 2021). The taxonomy of each 16S rRNA gene sequence was analyzed using RDP Classifier (version 2.2, http://sourceforfe.net/projects/rdp-classifier/, accessed on 1 December 2021) against the Silva 16S rRNA database [24]. Spearman’s correlations were presented by using R packages heatmap (R3.1.0).

### 2.6. Quantitative Analysis of Bile Acids in Mouse Liver

Bile acids in the liver of mice were quantified by LC/MS. The separation of bile acid was conducted using an Acquity UPLC BEH C18 column (2.1 × 100 mm, 1.7 μm) (Waters, Milford, MA, USA). The sample injection volume was 5 μL, with a column temperature at 40 °C. The mobile phase consisted of formic acid (0.01% in water, solvent A) and acetonitrile (solvent B), at a flow rate of 0.25 mL/min. The gradient elution program was as follows: 0–4 min, 25% B; 4–9 min, 25–30% B; 9–14 min, 30–36% B; 14–18 min, 36–38% B; 18–24 min, 38–50% B; 24–32 min, 50–75% B; 32–35 min, 75–100% B; and 35–38 min, 100–25% B. The samples were analyzed by a mass spectrometer in negative electrospray ionization under a multiple reaction mode (MRM). Operating parameters were temperature, 500 °C; ion spray voltage, −4500 V; curtain gas, 30 psi; collision gas, 6 psi; ion source gas 1, 50 psi; ion source gas 2, 50 psi.

### 2.7. Statistical Analysis

Data were presented as the mean ± SEM. Statistical significance of difference was determined with a one-way ANOVA analysis, followed by Duncan’s multiple range test. All statistical analyses were calculated with SPSS 20.0 software (version 23.0, IBM Inc., Chicago, IL, USA).

## 3. Results

### 3.1. Synbiotic Yogurt Enriched with Inulin Reduces Weight Gain and Improves the Serum Hormones Profiles in PCOS Mice

**Figure 1:**
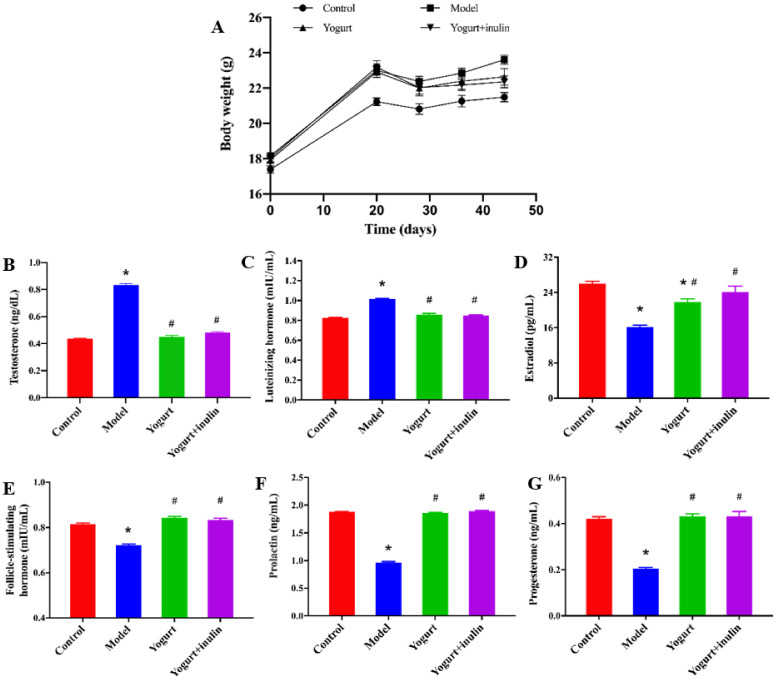
Effect of synbiotic yogurt on body weight and the levels of serum sex hormones in PCOS mice: (**A**) body weight; (**B**) serum testosterone levels (**C**) serum luteinizing hormone levels; (**D**) serum estradiol levels; (**E**) serum follicle-stimulating hormone levels; (**F**) serum prolactin levels; (**G**) serum progesterone levels. Data are expressed as the mean ± SEM (*n* = 6). * *p* < 0.05 vs. control group, ^#^ *p* < 0.05 vs. model group.

As shown in Figure 1A, the body weight showed no difference among the four groups before the injection of DHEA. After 20 days of DHEA treatment, the body weight of DHEA-treated mice was increased significantly, compared with the control group, while yogurt- and synbiotic-yogurt-containing inulin treatment significantly lowered the body weight, compared with that of DHEA-treated mice in the model group, and the effect of synbiotic yogurt was more obvious than that of yogurt (Figure 1A).

Next, the serum hormones associated with ovarian function including E2, T, LH, FSH, prolactin PRL, and PROG were measured. The serum levels of total T (Figure 1B) and LH (Figure 1C) in PCOS mice were significantly higher than those in the control mice. Yogurt and synbiotic yogurt decreased the serum levels of total T and LH. The levels of E2 (Figure 1D), PROG (Figure 1E), PRL (Figure 1F), and FSH (Figure 1G) in serum were significantly lower in the model group, while yogurt and synbiotic yogurt apparently up-regulated these hormone levels in serum.

### 3.2. Synbiotic Yogurt Enriched with Inulin Improves Ovary Morphology and Estrous Cycle in PCOS Mice

**Figure 2:**
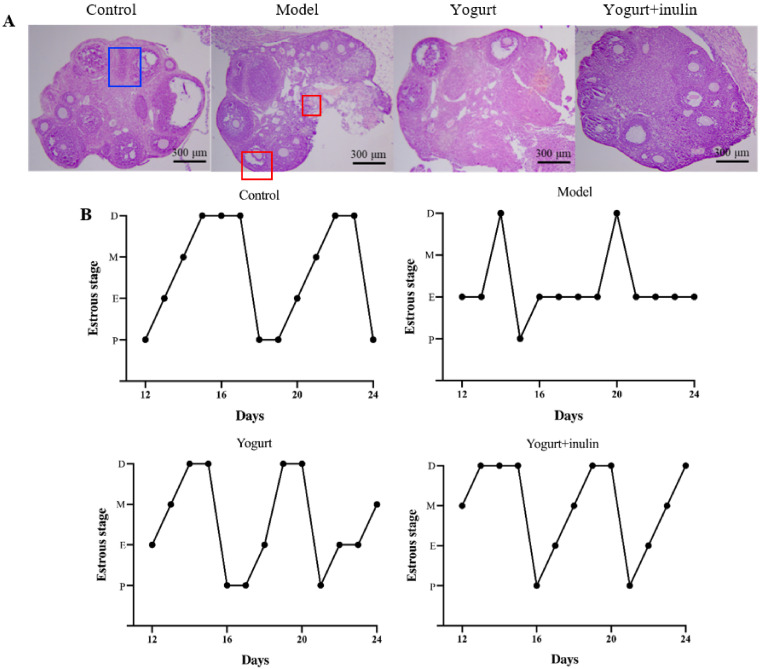
Effect of synbiotic yogurt on estrous cycle and ovarian morphology in PCOS mice: (**A**) H&E staining analysis of ovarian morphology. Bar = 300 μm. The cystic follicle is marked by red blocks, while the corpora lutea is marked by a blue block; (**B**) estrous cycle. M: metestrus, E: estrus, P: proestrus, D: diestrus. Data are expressed as the mean ± SEM (*n* = 5).

The histological analysis of ovaries showed normal features with multiple follicles at different development stages in the control group. PCOS ovaries lacked corpora lutea and contained multiple cystic follicles, followed by the attenuation of granulosa cell layers. Treatment with yogurt or synbiotic yogurt reduced the number of cystic follicles and increased the number of corpora lutea in PCOS mice. Moreover, synbiotic yogurt supplemented with inulin had a stronger effect than yogurt (Figure 2A), suggesting a therapeutic role of synbiotic yogurt in PCOS mice.

The vaginal cytological examination showed that the phases of the estrus cycle followed the regular changes in the order of proestrus, estrus, metestrus, and diestrus in the normal group (Figure 2B). The estrous cycle of the model group was disordered, as shown by the disappearance of the diestrus period and the prolongation of the estrous period. However, the intervention of yogurt and synbiotic yogurt partially improved estrus cycles in PCOS mice.

### 3.3. Synbiotic Yogurt Enriched with Inulin Improves the Serum Levels of Immune Cell-Produced Cytokines in PCOS Mice

**Figure 3:**
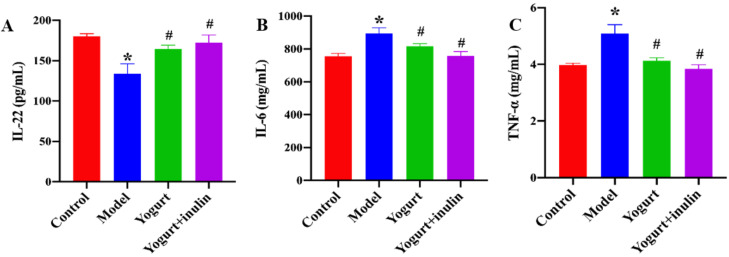
Effect of synbiotic yogurt on the levels of serum inflammatory cytokines in PCOS mice: (**A**) serum IL-22 levels; (**B**) serum IL-6 levels; (**C**) serum TNF-a levels. Data are expressed as the mean ± SEM (*n* = 6). * *p* < 0.05 vs. control group, ^#^ *p* < 0.05 vs. model group.

The serum levels of immune cell-produced cytokines including IL-6, IL-22, and TNF-α were also determined. PCOS mice showed a lower level of IL-22 (Figure 3A) and higher levels of IL-6 (Figure 3B) and TNF-α (Figure 3C). However, the treatment of yogurt and synbiotic yogurt supplemented with inulin significantly increased the levels of IL-22 while decreasing the levels of IL-6 and TNF-α.

### 3.4. Synbiotic Yogurt Enriched with Inulin Regulates the Composition of Gut Microbiota in PCOS Mice

**Figure 4:**
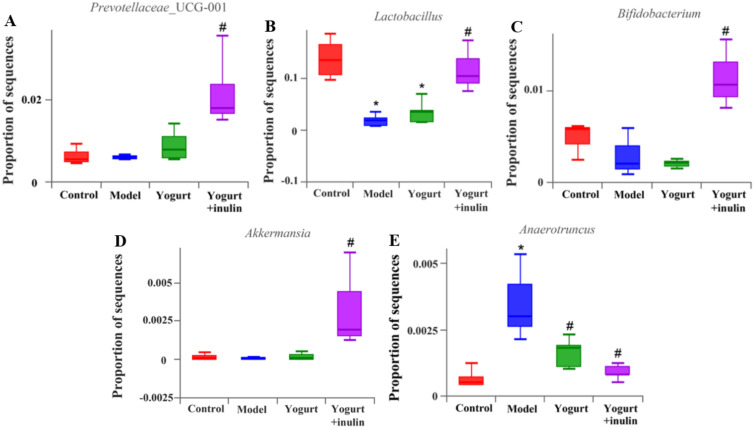
Effect of synbiotic yogurt on the composition of gut microbiota in PCOS mice at the genus level. Relative abundance of *Prevotellaceae*_UCG-001 (**A**), *Lactobacillus* (**B**), *Bifidobacterium* (**C**), *Akkermansia* (**D**), and *Anaerotruncus* (**E**). Data are expressed as mean ± SEM (*n* = 6). * *p* < 0.05 vs. control group, ^#^ *p* < 0.05 vs. model group.

At the genus level, the relative abundance of *Lactobacillus* (Figure 4B) and *Bifidobacterium* (Figure 4C) was decreased, and the relative abundance of *Anaerotruncus* (Figure 4E) was increased in the model group, compared with the control group. The administration of synbiotic-yogurt-containing inulin increased the relative abundance of *Lactobacillus* and *Bifidobacterium* by 6.65 fold and 3.92 fold, respectively, and suppressed the abundance of *Anaerotruncus* by 75.71%, compared with the model group. Additionally, compared with the model group, synbiotic yogurt treatment also increased the relative abundance of *Prevotellaceae*_UCG-001(3.70 fold) (Figure 4A) and *Akkermansia* (59.29 fold) (Figure 4D) in PCOS mice.

### 3.5. PICRUSt Analysis and Prediction of Genomic Functional Changes

**Figure 5:**
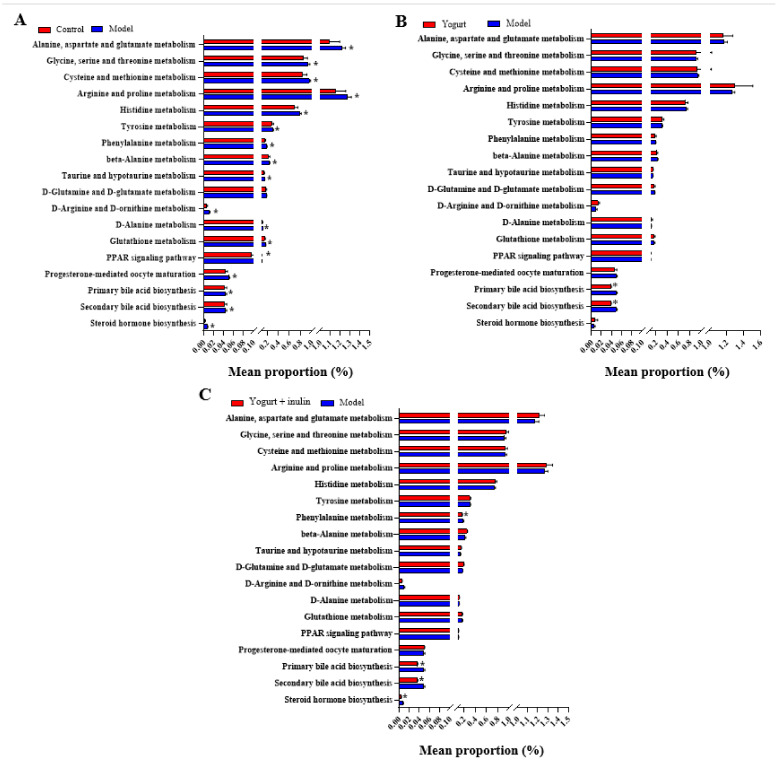
Functional prediction of changed KEGG pathways by PICRUSt. The altered KEGG pathways between control group and model group (**A**), between model group and yogurt group (**B**), and between model group and synbiotic yogurt group (**C**). * *p* < 0.05.

To further explore the effects of synbiotic yogurt supplemented with inulin on the gut microbiota, hormone levels, and metabolism, the functional gene profiles of bacterial communities were predicted by PICRUSt analysis. The predicted functions were blasted with the database of the KEGG pathway. The KEGG pathway (level 3) compositions in bacterial populations were analyzed, as shown in Figure 5. KEGG pathway analysis showed that there were significant differences in progesterone-mediated oocyte maturation, PPAR signaling pathway, primary bile acid biosynthesis, secondary bile acid biosynthesis, and steroid hormone biosynthesis between the control group and model group. Compared with the model group, the gene abundances in pathways of bile acid biosynthesis (primary bile acid biosynthesis and secondary bile acid biosynthesis) were decreased after yogurt supplementation. Moreover, synbiotic-yogurt-containing inulin treatment decreased the gene abundances in the pathways of bile acid biosynthesis (primary bile acid biosynthesis and secondary bile acid biosynthesis) and steroid hormone biosynthesis. These results suggest that the shifts of gut bacterial functional profiles responding to gut community changes induced by synbiotic yogurt might be related to the improvement of bile acid biosynthesis and steroid hormone biosynthesis in PCOS mice.

### 3.6. Synbiotic Yogurt Enriched with Inulin Regulates the Composition of Gut Microbiota in PCOS Mice

**Figure 6:**
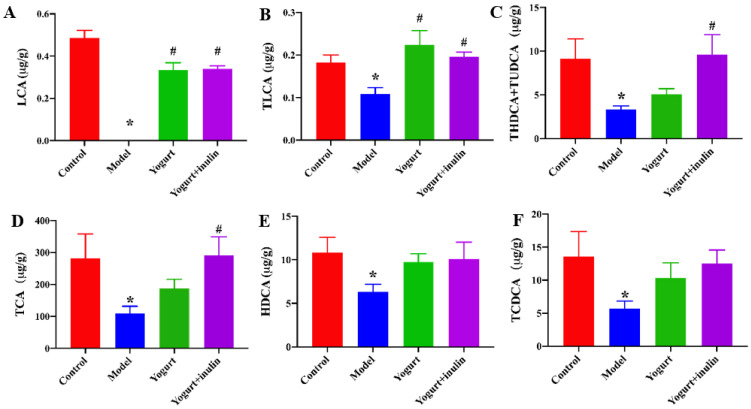
Effect of synbiotic yogurt on the bile acid profiles in the liver of PCOS mice. The levels of lithocholic acid (LCA) (**A**), taurolithocholic acid sodium salt (TLCA) (**B**), taurohyodeoxycholic acid sodium salt + tauroursodeoxycholic acid sodium salt (THDCA + TUDCA) (**C**), taurocholic acid sodium salt (TCA) (**D**), hyodeoxycholic acid (HDCA) (**E**), and taurochenodeoxycholic acid (TCDCA) (**F**) were detected in PCOS mice. * *p* < 0.05 vs. control group, ^#^ *p* < 0.05 vs. model group.

To further investigate the effects of synbiotic yogurt enriched with inulin on bile acid biosynthesis in PCOS mice, the bile acid profiles in the liver were measured by LC/MS. As shown in Figure 6, compared with the control group, PCOS mice showed lower levels of lithocholic acid (LCA), taurolithocholic acid sodium salt (TLCA), taurohyodeoxycholic acid sodium salt + tauroursodeoxycholic acid sodium salt (THDCA + TUDCA), taurocholic acid sodium salt (TCA), hyodeoxycholic acid (HDCA), and taurochenodeoxycholic acid (TCDCA). Yogurt treatment significantly increased the concentrations of LCA and TLCA, and synbiotic yogurt increased the concentrations of LCA, TLCA, THDCA + TUDCA, and TCA in PCOS mice.

### 3.7. Correlation between Gut Microbiota and Bile Acid Profiles

**Figure 7:**
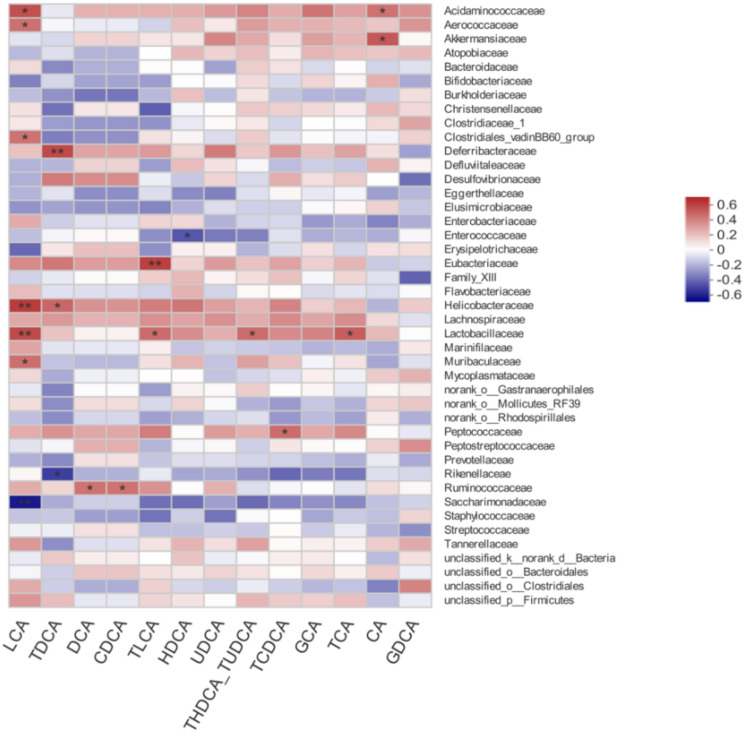
Spearman’s correlation between gut microbiota and bile acid profiles. * *p* < 0.05, ** *p* < 0.01.

The correlations between gut microbiota and bile acid profiles were also calculated by Spearman’s rho non-parametric correlation analysis (Figure 7). Results showed that *Acidaminococcaceae* was positively associated with LCA and CA. *Aerococcaceae*, *Clostridiales_vadinBB60_group,* and *Muribaculaceae* were positively associated with LCA. *Akkermansiaceae* was positively associated with CA. *Deferribacteraceae* had a positive relation with TDCA. *Enterococcaceae* had a negative relation with HDCA. *Eubacteriaceae* had a positive relation with TLCA. *Helicobacteraceae* had a positive relation with LCA and TDCA. The heatmap also reflected significant positive correlations between *Lactobacillaceae* and LCA, TLCA, THDCA + TUDCA, and TCA.

## 4. Discussion

Common to all classifications, the diagnosis of PCOS requires a minimum of two of the following items including hyperandrogenism, oligoanovulation, and polycystic ovaries [2]. PCOS can also increase the risk of obesity, diabetes, and metabolic syndrome [7]. A PCOS-mediated metabolic disorder is related to hyperandrogenism, which occurs regardless of body mass index [4]. Since evaluating the etiology of PCOS and obtaining ovarian tissue from patients are difficult, PCOS models induced by DHEA have been developed to investigate different aspects of its pathogenesis [25]. DHEA-induced PCOS mice exhibit many common features with PCOS individuals, such as hyperandrogenism, abnormal maturation of ovarian follicles, anovulation, and insulin resistance [26]. In addition, DHEA-induced PCOS mice also have symptoms of infertility, accompanied by an increase in the number of atretic follicles and follicular cysts in the ovary [27]. Consistent with these findings, in our study, DHEA treatment for 20 days increased the body weight gain, disrupted the estrous cycle, and increased the number of multiple cystic follicles in ovaries, indicating that the PCOS mouse model mimics PCOS symptoms in human patients.

Follicular development occurs under the influence of androgen hormones [28]. The androgen hormones are produced by the ovary, which can be described by the classical two-cells–two-hormones model [28]. In the theca cells, LH stimulates the production of androgens (androstenedione and testosterone). Then, the androgens are converted to estrogens (estradiol and estrone) by the action of aromatase in response to the stimulation of FSH in granulosa cells [28]. In anovulatory women with PCOS, abnormal gonadotropic derangements occur due to an increase in GnRH pulse frequency, which contributes to the increase in LH levels and the reduction in FSH concentrations in serum [29]. The increased LH/FSH ratio in the ovaries further increased the circulating androgen level that impairs follicular development [30]. In our study, DHEA-induced PCOS mice also showed higher levels of LH and T and lower levels of E2, PROG, PRL, and FSH in serum, suggesting the disturbance of sex hormones. Only a few studies have explored the influence of probiotics or synbiotic treatment on PCOS and its related metabolic disorders. Resistant dextrin (a prebiotic) intervention for six months reduced the body weight in PCOS patients [1]. Intake of *Lactobacillus* acidophilus Strain T16, *Lactobacillus casei* Strain T2, and *Bifidobacterium bifidum* Strain T1 plus inulin improved the metabolic status, accompanied by the reduction in serum levels of insulin and triglycerides, in PCOS individuals [16]. Another study showed that inulin decreased the body weight and the T levels, increased E2 levels, and ameliorated PCOS symptoms in PCOS mice [21]. In our study, synbiotic yogurt also reduced T levels and increased the levels of E2, PROG, PRL, and FSH in serum.

Recently, several studies have shown a connection between gut microbiota and PCOS. The disturbances in gut microbiota, often referred to as “dysbiosis of the gut microbiota”, promote intestinal permeability with the massive release of lipopolysaccharide (LPS) produced by Gram-negative bacteria into the bloodstream, triggering a chronic inflammatory response [6]. A meta-analysis study suggested that women with PCOS showed higher concentrations of pro-inflammatory factors including IL-6, which may serve as monitoring biomarkers for the treatment of PCOS [31]. Our study indicated a significant reduction in IL-6 and TNF-α in PCOS mice after synbiotic yogurt treatment. In addition, the resultant hyperinsulinemia induced by the chronic inflammatory response drives the excess production of androgen and impairs follicular development, leading to PCOS [6]. Many species of gut microbiota, especially *Bifidobacteria* and *Lactobacillus*, exert health-promoting properties, which are generally considered to be “good” or “beneficial” bacteria [32]. The treatment of *Lactobacillus* and fecal microbiota transplantation to PCOS rats decreased the levels of androgens and restored the estrous cycles, thus improving the ovarian functions [33]. Probiotic *Bifidobacterium lactis* V9 modulated the gut microbiome and regulated the secretion of sex hormones in women of PCOS [34]. A study showed that PCOS women or PCOS rats exhibited lower *Bifidobacterium* and *Lactobacillus* [34], and we observed a decrease in the relative abundance of *Bifidobacterium* in the model group, compared with the control group. However, in our study, synbiotic treatment increased the relative abundance of *Bifidobacterium* and *Lactobacillus* in PCOS mice, which was in accordance with reports about the role of inulin on the gut microbiota of PCOS mice [21]. PCOS patients showed a reduction in the abundance of *Akkermansia* regardless of obesity [7]. In our study, after treatment of synbiotic, PCOS mice became enriched with *Akkermansia*, while no difference was observed between the control group and model group. Consistent with this finding, metformin administration to PCOS women increased the relative abundance of *Akkermansia* [35]. These results suggest *Akkermansia* may have a beneficial effect in combating PCOS-related metabolic disorders.

The bile acid metabolism pathway is a potential mechanism between the gut microbiome and PCOS [12]. Gut microbiota have a variety of metabolic functions, including the capacity to synthetize, metabolize, and reabsorb bile acids [36]. Quantitative profiling of bile acids found that the levels of secondary bile acids including GDCA and TUDCA were markedly decreased in serum and feces of PCOS women, compared with those in healthy women [12]. Metabolomics analysis showed that PCOS women had a lower level of glycocholic acid in serum, suggesting that the lipid absorption becomes disordered [37]. In our study, the concentrations of several bile acids including LCA, TLCA, and TCA were also decreased in PCOS mice, which was reversed by synbiotic supplementation. A recent study demonstrated that *Bacteroides vulgatus* abundance was markedly elevated in PCOS patients, accompanied by the reduction in glycodeoxycholic acid levels. Mechanistically, glycodeoxycholic acid activated GATA3, to induce the excretion of IL-22, which further mitigated PCOS symptoms, suggesting the regulatory effect of gut microbiota–bile acid-IL-22 axis on the etiology of PCOS [12]. In accordance with this finding, correlation analysis in our study showed that many specific bacteria, including *Akkermansiaceae* and *Lactobacillaceae*, were associated with bile acid profiles. Moreover, the serum level of IL-22 was lower in PCOS mice, compared with the control group, which was also reversed by synbiotic yogurt treatment. These suggest that the regulation of gut microbiota by synbiotic may further improve the bile acid profiles. As signaling molecules, bile acids can also bind and activate receptors in the pathway of G protein-coupled bile acid receptor (TGR5), farnesoid X receptor (FXR), and vitamin D receptor (VDR) [9]. Therefore, the contributions of gut bacteria that can deconjugate bile acids, and the influence and mechanism of synbiotic yogurt on the specific bile acids in PCOS remain unclear and need to be further investigated.

## 5. Conclusions

Through 24-day yogurt treatment of PCOS mice, yogurt supplemented with inulin decreased the body weight gain, improved the sex hormones and pro-inflammatory factors profiles, and alleviated ovarian dysfunction. Inulin-enriched synbiotic yogurt also regulated gut microbiota composition, along with the improvement in bile acid profiles, suggesting that synbiotic yogurt treatment displayed potential for prevention and therapy of PCOS.

## Data Availability

All data supporting the findings of this study are available within the figures. Raw data are available on request from the corresponding author.

## References

[1] Shamasbi S.G., Dehghan P., Mohammad-Alizadeh Charandabi S., Aliasgarzadeh A., Mirghafourvand M. (2018). Effect of prebiotic on anthropometric indices in women with polycystic ovarian syndrome: A triple-blind, randomized, controlled clinical trial. Iran. Red Crescent Med. J..

[2] Escobar-Morreale H.F. (2018). Polycystic ovary syndrome: Definition, aetiology, diagnosis and treatment. Nat. Rev. Endocrinol..

[3] Zhang S., Tu H., Zhu J., Liang A., Huo P., Shan K., He J., Zhao M., Chen X., Lei X. (2020). Dendrobium nobile Lindl. polysaccharides improve follicular development in PCOS rats. Int. J. Biol. Macromol..

[4] Goodarzi M.O., Dumesic D.A., Chazenbalk G., Azziz R. (2011). Polycystic ovary syndrome: Etiology, pathogenesis and diagnosis. Nat. Rev. Endocrinol..

[5] Barko P.C., McMichael M.A., Swanson K.S., Williams D.A. (2018). The gastrointestinal microbiome: A review. J. Vet. Intern. Med..

[6] Tremellen K., Pearce K. (2012). Dysbiosis of Gut Microbiota (DOGMA)—A novel theory for the development of polycystic ovarian syndrome. Med. Hypotheses.

[7] Liu R., Zhang C., Shi Y., Zhang F., Li L., Wang X., Ling Y., Fu H., Dong W., Shen J. (2017). Dysbiosis of gut microbiota associated with clinical parameters in polycystic ovary syndrome. Front. Microbiol..

[8] Torres P.J., Siakowska M., Banaszewska B., Pawelczyk L., Duleba A.J., Kelley S.T., Thackray V.G. (2018). Gut microbial diversity in women with polycystic ovary syndrome correlates with hyperandrogenism. J. Clin. Endocrinol. Metab..

[9] McGlone E.R., Bloom S.R. (2019). Bile acids and the metabolic syndrome. Ann. Clin. Biochem..

[10] Wahlstrom A., Sayin S.I., Marschall H.U., Backhed F. (2016). Intestinal crosstalk between bile acids and microbiota and its impact on host metabolism. Cell Metab..

[11] Li C., Li Y., Gai Z. (2019). Bile acids and farnesoid X receptor: Novel target for the treatment of diabetic cardiomyopathy. Curr. Protein Pept. Sci..

[12] Qi X., Yun C., Sun L., Xia J., Wu Q., Wang Y., Wang L., Zhang Y., Liang X., Wang L. (2019). Gut microbiota-bile acid-interleukin-22 axis orchestrates polycystic ovary syndrome. Nat. Med..

[13] Zhang B., Shen S., Gu T., Hong T., Liu J., Sun J., Wang H., Bi Y., Zhu D. (2019). Increased circulating conjugated primary bile acids are associated with hyperandrogenism in women with polycystic ovary syndrome. J. Steroid Biochem. Mol. Biol..

[14] Million M., Angelakis E., Paul M., Armougom F., Leibovici L., Raoult D. (2012). Comparative meta-analysis of the effect of Lactobacillus species on weight gain in humans and animals. Microb. Pathog..

[15] Suzumura E.A., Bersch-Ferreira A.C., Torreglosa C.R., Da Silva J.T., Coqueiro A.Y., Kuntz M.G.F., Chrispim P.P., Weber B., Cavalcanti A.B. (2019). Effects of oral supplementation with probiotics or synbiotics in overweight and obese adults: A systematic review and meta-analyses of randomized trials. Nutr. Rev..

[16] Samimi M., Dadkhah A., Haddad Kashani H., Tajabadi-Ebrahimi M., Seyed Hosseini E., Asemi Z. (2019). The effects of synbiotic supplementation on metabolic status in women with polycystic ovary syndrome: A randomized double-blind clinical trial. Probiotics Antimicrob. Proteins.

[17] Rashad N.M., El-Shal A.S., Amin A.I., Soliman M.H. (2017). Effects of probiotics supplementation on macrophage migration inhibitory factor and clinical laboratory feature of polycystic ovary syndrome. J. Funct. Foods.

[18] Sun Q., Zhu L., Li Y., Cui Y., Jiang S., Tao N., Chen H., Zhao Z., Xu J., Dong C. (2020). A novel inulin-type fructan from Asparagus cochinchinensis and its beneficial impact on human intestinal microbiota. Carbohydr. Polym..

[19] Dewulf E.M., Cani P.D., Claus S.P., Fuentes S., Puylaert P.G., Neyrinck A.M., Bindels L.B., De Vos W.M., Gibson G.R., Thissen J.P. (2013). Insight into the prebiotic concept: Lessons from an exploratory, double blind intervention study with inulin-type fructans in obese women. Gut.

[20] Zou J., Chassaing B., Singh V., Pellizzon M., Ricci M., Fythe M.D., Kumar M.V., Gewirtz A.T. (2018). Fiber-mediated nourishment of gut microbiota protects against diet-induced obesity by restoring IL-22-mediated colonic health. Cell Host Microbe.

[21] Xue J., Li X., Liu P., Li K., Sha L., Yang X., Zhu L., Wang Z., Dong Y., Zhang L. (2019). Inulin and metformin ameliorate polycystic ovary syndrome via anti-inflammation and modulating gut microbiota in mice. Endocr. J..

[22] Liu H., Liu M., Fu X., Zhang Z.Q., Zhu L.Y., Zheng X., Liu J.S. (2018). Astaxanthin prevents alcoholic fatty liver disease by modulating mouse gut microbiota. Nutrients.

[23] Caporaso J.G., Kuczynski J., Stombaugh J., Bittinger K., Bushman F.D., Costello E.K., Fierer N., Pena A.G., Goodrich J.K., Gordon J.I. (2010). QIIME allows analysis of high-throughput community sequencing data. Nat. Methods.

[24] Quast C., Pruesse E., Yilmaz P., Gerken J., Schweer T., Yarza P., Peplies J., Glockner F.O. (2012). The SILVA ribosomal RNA gene database project: Improved data processing and web-based tools. Nucleic Acids Res..

[25] Zhu J.Q., Zhu L., Liang X.W., Xing F.Q., Schatten H., Sun Q.Y. (2010). Demethylation of LHR in dehydroepiandrosterone-induced mouse model of polycystic ovary syndrome. Mol. Hum. Reprod..

[26] Luchetti C.G., Solano M.E., Sander V., Arcos M.L., Gonzalez C., Di Girolamo G., Chiocchio S., Cremaschi G., Motta A.B. (2004). Effects of dehydroepiandrosterone on ovarian cystogenesis and immune function. J. Reprod. Immunol..

[27] Li S.Y., Song Z., Song M.J., Qin J.W., Zhao M.L., Yang Z.M. (2016). Impaired receptivity and decidualization in DHEA-induced PCOS mice. Sci. Rep..

[28] Gervasio C.G., Bernuci M.P., Silva-de-Sa M.F., Rosa E.S.A.C. (2014). The role of androgen hormones in early follicular development. ISRN Obstet. Gynecol..

[29] Orisaka M., Hattori K., Fukuda S., Mizutani T., Miyamoto K., Sato T., Tsang B.K., Kotsuji F., Yoshida Y. (2013). Dysregulation of ovarian follicular development in female rat: LH decreases FSH sensitivity during preantral-early antral transition. Endocrinology.

[30] Solorzano C.M.B., McCartney C.R., Blank S.K., Knudsen K.L., Marshall J.C. (2010). Hyperandrogenaemia in adolescent girls: Origins of abnormal gonadotropin-releasing hormone secretion. BJOG Int. J. Obstet. Gynaecol..

[31] Peng Z., Sun Y., Lv X., Zhang H., Liu C., Dai S. (2016). Interleukin-6 levels in women with polycystic ovary syndrome: A systematic review and meta-analysis. PLoS ONE.

[32] Roberfroid M., Gibson G., Hoyles L., McCartney A., Rastall R., Rowland I., Wolvers D., Watzl B., Szajewska H., Stahl B. (2010). Prebiotic effects metabolic and health benefits. Br. J. Nutr..

[33] Guo Y., Qi Y., Yang X., Zhao L., Wen S., Liu Y., Tang L. (2016). Association between polycystic ovary syndrome and gut microbiota. PLoS ONE.

[34] Zhang J., Sun Z., Jiang S., Bai X., Ma C., Peng Q., Chen K., Chang H., Fang T., Zhang H. (2019). Probiotic *Bifidobacterium lactis* V9 regulates the secretion of sex hormones in polycystic ovary syndrome patients through the gut-brain axis. mSystems.

[35] Shin N.R., Lee J.C., Lee H.Y., Kim M.S., Whon T.W., Lee M.S., Bae J.W. (2014). An increase in the *Akkermansia* spp. population induced by metformin treatment improves glucose homeostasis in diet-induced obese mice. Gut.

[36] Festi D., Schiumerini R., Eusebi L.H., Marasco G., Taddia M., Colecchia A. (2014). Gut microbiota and metabolic syndrome. World J. Gastroenterol..

[37] Jia C., Xu H., Xu Y., Xu Y., Shi Q. (2019). Serum metabolomics analysis of patients with polycystic ovary syndrome by mass spectrometry. Mol. Reprod. Dev..

